# *Clostridium haemolyticum* Infection: A Cause of Hemolytic Anemia in a Patient with Bone Marrow Necrosis

**DOI:** 10.3390/microorganisms9081568

**Published:** 2021-07-23

**Authors:** Anne Sophie Lagneaux, Sandrine Hénard, Laure Diancourt, Emmanuelle Stein, Pierre Perez, Pierre Mathieu, Corentine Alauzet, Alain Lozniewski

**Affiliations:** 1 Service de Microbiologie, CHRU de Nancy, 54500 Vandœuvre-lès-Nancy, France; a.lagneaux@chru-nancy.fr (A.S.L.); c.alauzet@chru-nancy.fr (C.A.); 2 Service des Maladies Infectieuses et Tropicales, CHRU de Nancy, 54500 Vandœuvre-lès-Nancy, France; s.henard@chru-nancy.fr; 3 CNR Bactéries Anaérobies et Botulisme, Institut Pasteur, 75015 Paris, France; laure.diancourt@pasteur.fr; 4 Laboratoire de Biologie Médicale, CH de Verdun–Saint Mihiel, 55100 Verdun, France; estein@ch-verdun.fr (E.S.); pmathieu@ch-verdun.fr (P.M.); 5 Service de Médecine Intensive et Réanimation, Hôpitaux de Brabois, CHRU de Nancy, 54500 Vandœuvre-lès-Nancy, France; p.perez@chru-nancy.fr; 6 Laboratoire SIMPA Stress Immunité Pathogènes UR 7300, Service de Microbiologie, Université de Lorraine, CHRU de Nancy, 54500 Vandœuvre-lès-Nancy, France

**Keywords:** *Clostridium haemolyticum*, hemolytic anemia, bone marrow necrosis, human infection

## Abstract

*Clostridium haemolyticum* is a sporulating Gram-positive anaerobic rod that is considered to be one of the most fastidious and oxygen-sensitive anaerobes. It is a well-known animal pathogen and the cause of bacillary hemoglobinuria primarily in cattle. To date, human infections caused by *C. haemolyticum* have been reported in three patients with malignant underlying diseases. We present herein the case of a 30-year-old obese woman with no significant past medical history who developed bacteremia caused by *C. haemolyticum* with massive intravascular hemolysis associated with bone marrow necrosis and acute renal failure. Because of subculture failure, the diagnosis was made on the basis of 16S rDNA sequencing and next-generation sequencing. The patient, who had been afebrile for 20 days after a 17-day-course of antibiotics, experienced a second bacteremic episode caused by *C. haemolyticum*. After having been successfully treated for 42 days with clindamycin and amoxicillin-clavulanic acid, the patient developed acute myeloid leukemia as a result of bone marrow regeneration. Although uncommon in humans, infections caused by *C. haemolyticum* are severe and should be considered in a febrile patient who has severe hemolytic anemia. This case also highlights the importance of using molecular techniques for the identification of this fastidious anaerobic organism.

## 1. Introduction

*Clostridium haemolyticum* is an anaerobic, motile, spore-forming Gram-positive rod [1]. This species belongs to the *Clostridium novyi sensu lato* group, which also includes two other genetically closely related species, *Clostridium novyi* and *Clostridium botulinum* (group III) [1,2,3].

*C. haemolyticum* is considered to be one of the most fastidious and oxygen-sensitive anaerobes known [1]. Thus, culture of this organism is very difficult and requires the use of freshly prepared pre-reduced media and rigorous anaerobiosis during incubation and manipulations. Under these conditions, small gray colonies (1–3 mm in diameter) with zones of hemolysis may be seen on blood agar [1,4]. Similar to some other clostridial organisms, it may appear Gram negative in culture. Spores are oval, subterminal, and swell the cell.

In animals, *C. haemolyticum* is the cause of a rapidly fatal disease known as bacillary hemoglobinuria or red-water disease [5]. Bacillary hemoglobinuria mainly affects cattle but also, more rarely, other animals, such as sheep, horses, buffaloes, hogs and elks [6,7,8,9,10,11,12,13,14].

Although not fully understood, the virulence of *C. haemolyticum* is mainly attributed to the production of exotoxins such as the beta, eta and theta toxins [1]. It has also been suggested that *C. haemolyticum* proteolytic flagellins might facilitate tissue spreading and necrosis [15]. Among exotoxins produced by this organism, the beta toxin, which is a phospholipase that cleaves phosphatidylcholine by hydrolysis into phosphocholine and a diglyceride, is considered the main factor that contributes to the pathogenesis of bacillary hemoglobinuria [1,5,16]. This toxin lyses erythrocytes and hepatocytes, and damages capillary endothelium. Considering the action of closely related phospholipases C, it has also been suggested that the beta toxin of *C. haemolyticum* may activate the arachidonic acid cascade in eukaryotic cells, which may in turn induce inflammation, platelet aggregation and increased vascular permeability [5,17].

Spores of *C. haemolyticum* are found in soils where they may survive for several years. Once ingested, spores may reach the liver and more rarely other tissues, including kidneys and bone marrow, where they may remain latent within local phagocytes [5]. In animals, the occurrence of bacillary hemoglobinuria follows the germination of spores present in the resident macrophages of the liver (Kupffer cells). The vegetative forms then produce potent toxins, mainly the beta toxin, which causes the disruption of Kupffer cells. Once released, the beta toxin induces hepatocellular necrosis as well as intravascular hemolysis, leading to anemia and hemoglobinuria [5].

To date, only three cases of human infection caused by *C. haemolyticum* have been reported [18,19,20]. We report herein a new case of *C. haemolyticum* infection that has occurred in a woman with bone marrow necrosis and acute myeloid leukemia.

## 2. Case Presentation

A 30-year-old obese woman with no significant past medical history was admitted (day 0) to the Emergency Department of the Hospital of Verdun, France, for one-week persistent low back pain, fever at 39 °C, and 48 h hematuria. Plasma and urine were both dark red, suggesting massive hemolysis. This was confirmed by laboratory investigations that revealed hemolytic anemia with a hemoglobin level of 4.5 g/dL, a mean corpuscular level of 86 fL, a reticulocyte count of 150 Giga/L, a LDH level of 13,000 U/L, and an unconjugated bilirubin level of 55 µmol/L. Direct and indirect Coombs tests were negative, while red blood cell morphology was normal on blood smear. Laboratory tests also showed a marked inflammatory response: hyperleukocytosis (leukocytes 19.3 Giga/L) with elevated neutrophils (12 Giga/L) and increased blood levels of C-reactive protein (47 mg/L) and procalcitonin (7.78 ng/mL). No blast cells were detected on blood smear. A computed tomography of the chest, abdomen and pelvic area was unremarkable except for mild splenomegaly. Two sets of blood cultures were sampled and incubated for 5 days (BACT/ALERT system). The patient was empirically treated with cefotaxime IV and received red blood cell transfusion. She was then transferred (day 1) to the University Hospital of Nancy, France.

**Figure 1:**
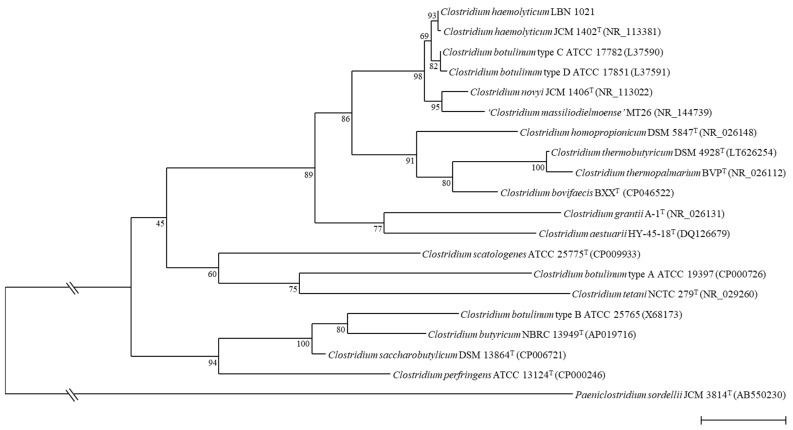
Maximum likelihood (computed by PHYML, model GTR plus gamma distribution and invariant sites) phylogenetic tree based on partial 16S rRNA gene sequences (1420 nt) showing relationships between the strain isolated in our patient (LBN 1021) and related clostridia species. The *Paeniclostridium sordellii* 16S rRNA gene sequence (AB550230) was used as the outgroup. Numbers at nodes indicate percentages of bootstrap support, based on analysis of 100 replicates. Species represented in single quotes are effectively published but not validly published. Bar, 0.02 substitutions per site.

On admission at this hospital, blood tests revealed a marked hepatic cytolysis with aspartate transaminase (AST) levels of 944 U/L and alanine transaminase (ALT) levels of 60 U/L, an increased white blood cells response (leukocytes 42 Giga/L) and a low platelet count (98 Giga/L) related to disseminated intravascular coagulation. Development of acute renal failure was also evidenced (creatinine: 42.4 mg/L). On that day, metronidazole IV was added to the cefotaxime due to the presence of sporulated Gram-variable bacilli in two anaerobic vials collected on day 0. All subcultures performed from positive blood culture vials on various enriched blood agar media remained sterile despite the use of pre-reduced media, work in an anaerobic chamber and prolonged incubation. After 72 h of subculture in pre-reduced Schaedler broth (SB), a moderate growth was detected. Microscopic examination of the broth revealed the presence of decolorized Gram-positive, spore-forming rods. While further subcultures of the positive broth on agar media and in SB remained negative, a broad range 16S rDNA PCR [21] performed on a DNA extract from SB led to a 651 bp sequence that showed 100% similarities with the sequence of the type strain JCM 1402^T^ of *Clostridium haemolyticum* (accession number AB640687). This sequence also shared 99.23% and 97.69% similarity with the sequence of *Clostridium botulinum* strain BKT015925 (accession number CP002410) and *Clostridium novyi* JCM 1406^T^ (accession number AB536772), respectively, with phylogenetic relationships with the closest clostridia species shown in Figure 1.

Taxonomic affiliation to the species *C. haemolyticum* was performed using Clinical and Laboratory Standards Institute interpretive criteria [22]. To confirm that our isolate belonged to the species *C. haemolyticum*, a DNA extract was sent to the National Center of Reference for Anaerobes (Pasteur Institute) for next-generation sequencing (NGS) analysis. Libraries were prepared using the TruSeq^®^ DNA library preparation kit (Illumina, San Diego, CA, USA) and the genome was sequenced using the NextSeq 500 sequencer (Illumina). Thisled to the obtention of 141 contigs corresponding to an assembled genome of 2628,654 bp (GenBank assembly accession: GCA_910822285.1). The average nucleotide identity (ANI) value between this genome and the genome of *C. haemolyticum* NCTC 9693 was 98.90%, which confirmed that our isolate belonged to the species *C. haemolyticum* [23]. Analysis of virulence factors showed a phospholipase C (beta toxin) presenting 99.50% similarities by blastx with the beta toxin of *C. haemolyticum* NCTC 9693 (KEI16223) with the absence of a C-terminal repeat pattern positioned just after the STOP codon. Genome analysis also showed the absence of the alpha toxin encoding gene and the presence of four CDS affiliated to flagellin genes arranged in tandem on the chromosome. Two shared 100% similarities by blastx with the flagellinolysin protein of *C. haemolyticum* (accession number WP_128609809) and the other two shared 100% similarities by blastx with flagellinolysin protein of *C. novyi* type B (accession number WP_039219681). No known antibiotic resistance genes have been detected in this genome by using the ResFinder database.

While the patient had become afebrile on day 4, she developed bicytopenia (hemoglobin level: 6 g/dL; platelet count: 28 Giga/L) on day 5. The leucocyte count was of 3.6 Giga/L (neutrophils: 2.2 Giga/L) without blast cells. Bone marrow aspirate revealed a hypocellular marrow without blastic infiltration and with extensive necrosis. Bone marrow biopsy examination confirmed extensive necrosis without any other morphological findings. Direct examination and culture of bone marrow samples were negative, as was the 16S rDNA PCR performed on these samples. Thus, after further detailed laboratory investigations that permitted to rule out other causes of bone marrow necrosis, the diagnosis of ischemic bone marrow necrosis probably caused by *C. haemolyticum* related exotoxemia was made.

**Figure 2:**
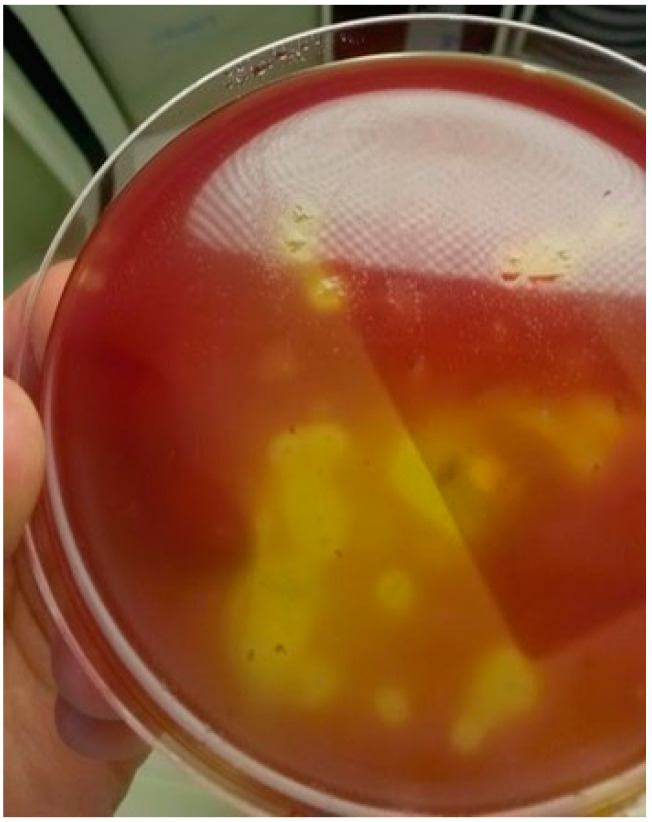
Colonies of *Clostridium haemolyticum* on fastidious anaerobe agar.

After 7 days of treatment with cefotaxime–metronidazole, the patient was treated with metronidazole IV for 3 days and then with amoxicillin IV for an additional 7 days. Since the patient was still apyretic, antibiotic therapy was stopped. At this time, hepatic cytolysis was no longer evidenced. However, because of a deterioration in her renal function (creatinine level: 76 mg/L), the patient underwent hemodialysis three times a week from day 13. On day 30, the patient, who had still a relatively well tolerated bicytopenia and was still apyretic, was transferred to the hospital center of Verdun. While her clinical condition was stable, she became feverish again on day 39. Three sets of blood cultures were sampled, and the patient was empirically treated with amoxicillin–clavulanic acid and clindamycin IV. After 24 h of incubation (BACT/ALERT system), one anaerobic bottle was positive with Gram-positive rods visualized by Gram stain microscopic examination. Broth blood aliquots of the positive blood culture were plated on a fastidious anaerobe agar (FAA) plate and inoculated in SB. All manipulations were performed inside an anaerobic chamber. After incubation in an anaerobic chamber, tiny β-hemolytic colonies were obtained on the FAA (Figure 2), while a slight growth was observed in SB.

Identification of the isolate (strain LBN 1021) from plates was performed using the Vitek MS and the Maldi Biotyper systems as previously described [24]. With the Vitek MS system (database version 2.0), the isolate remained unidentified either at the species or the genus level after direct deposit, on-plate formic acid treatment and ethanol–formic acid extraction. With the Biotyper system (IVD version, with 5627 entries), the isolate could not be identified after direct deposit, whereas a correct identification was achieved after ethanol–formic acid extraction. This identification was also confirmed by using a broad range 16S rDNA PCR, as previously mentioned, on a DNA extract from SB. Antibiotic susceptibility testing could not be performed since further subcultures remain negative despite manipulation in an anaerobic chamber and the use of pre-reduced media.

On day 43, the patient who was still bicytopenic was transferred to the University Hospital Center of Nancy. At that time, bone marrow aspirate analysis showed moderate regeneration with the presence of 18% atypical cells, while bone marrow biopsy analysis evidenced an infiltration by myeloid blasts. Further investigations permitted the confirmation of the diagnosis of acute myeloblastic leukemia M4. The patient was then treated for her leukemia while antibiotic therapy with clindamycin and amoxicillin was continued for a total of 42 days.

## 3. Discussion

In humans, only three cases of *C. haemolyticum* infections have been reported to date [18,19,20]. Son et al. [20] reported a case of liver abscess caused by *C. haemolyticum* that occurred after transarterial chemoembolization in a 76-year-old woman with hepatocellular carcinoma and diabetes mellitus. Saeb et al. [19] reported a case of bacteremia caused by *C. haemolyticum* in an 18-year-old patient who had a relapse of acute lymphoblastic leukemia after having a stem cell transplant two years before. Matsumoto et al. [18] reported a case where *C. haemolyticum* was the cause of a bone marrow infection in a patient treated by chemotherapy for a diffuse large B-cell lymphoma. It is noteworthy that in these cases, as in our case, patients who developed an infection caused by *C. haemolyticum* had underlying health problems. In previous reported cases, infection occurred in patients with malignant underlying conditions that may have contributed to the development of infection. In our case, *C. haemolyticum* was the cause of recurrent bacteremia in a patient who initially has no known underlying condition except obesity. The impact of this condition on the development of bacteremia caused by *C. haemolyticum* remains unclear. However, it cannot be ruled out considering the fact that obesity may cause various alterations of the immune system [25].

In the three previously reported human cases as well as in our case, the portal of entry of *C. haemolyticum* remained unknown. However, it may be speculated, on the basis of our knowledge of the pathogenesis of bacillary hemoglobinuria, that spores were ingested before being transported to the liver and/or to other tissues by lymphatics and blood. Spores are mainly found in areas with poorly drained pastures and alkaline pH, where they may survive for long periods [5]. In our case, it is noteworthy that the patient lived in a rural area where she might have been contaminated by the environment as she had no contact with cattle or other animals.

In animals, spores may remain latent within Kupffer cells for a long time and be converted into vegetative forms, which may cause, mainly via the production of exotoxins, damage to various cells. Beta toxin, which is considered to be the main factor involved in the virulence of *C. haemolyticum*, is a phospholipase C that induces hemolysis, hepatocyte and endothelial cell damage, platelet aggregation and increased vascular permeability [5]. Thus, as the disease progresses, the concentration of this exotoxin usually increases in the blood, leading to systemic consequences, including massive hemolytic anemia. In contrast to the other reported cases, it is noteworthy that our patient experienced, at the initial stage of disease, both important hepatic cytolysis and massive hemolytic anemia, which may be related to toxin-induced hepatocellular damage and further significant exotoxemia.

It has also been shown in animals that *C. haemolyticum* may reach the bone marrow by itself [26]. In the case reported by Matsumoto et al. [18], infection of the bone marrow by *C. haemolyticum* was also evidenced. In contrast, in our case, the fact that *C. haemolyticum* was not detected in bone marrow samples by culture or 16S rDNA PCR may suggest that ischemic bone marrow necrosis was most likely a consequence of multiple factors, including vascular damage induced by the beta toxin of *C. haemolyticum*, anemia-associated hypoxia and disseminated intravascular coagulation [27]. However, a bone marrow infection cannot be ruled out since bone marrow samples were taken after 5 days of antibiotic therapy. As observed in our case, renal failure may occur during bacillary hemoglobinuria [5]. In animals, renal failure is due to renal tubular necrosis, which is likely to be the consequence of anemia-associated hypoxia. However, in the present case, the etiology of renal failure remained unknown since a kidney biopsy could not be performed.

Our patient, who was afebrile after an initial 17-day-course of antibiotics, including β-lactams and metronidazole, experienced a second bacteremic episode caused by *C. haemolyticum* 20 days after the end of antibiotic therapy. The patient was then successfully treated by a 42-day-course of amoxicillin–clavulanate combined with clindamycin. The recurrence of bacteremia might be due to the persistence of spores in the digestive tract or within macrophages in the liver. However, this latter hypothesis is questionable considering the absence of hepatic cytolysis during the second bacteremic episode. Intracellular persisting organisms in other part of the body might also represent a reservoir for relapsing infection. Persistence of *C. haemolyticum* within the host might also have been favored by its resistance to the antibiotic used. It is noteworthy that no known antibiotic resistance genes were found in the genome of our isolate. However, the influence of antibiotic resistance in the recurrence of bacteremia may not be ruled out since no antibiotic susceptibility testing could be performed. Data on the antibiotic susceptibility of *C. haemolyticum* are scarce. According to these data, all strains tested to date were susceptible to beta-lactams, including aminopenicillins, and clindamycin, while one strain resistant to metronidazole was reported [4,18]. Thus, it cannot be ruled out that the recurrence of *C. haemolyticum* bacteremia was, at least partially, related to the persistence of organisms that were resistant to metronidazole.

*C. haemolyticum* is a fastidious organism that is considered one of the most oxygen-sensitive anaerobes. Thus, detection and identification of this pathogen is very difficult, as culture of this organism requires the use of adequate pre-reduced media and rigorous anaerobic conditions for incubation and manipulation of cultures. Despite the respect of these conditions, growth failure may occur, necessitating the use of molecular identification techniques. In our case, initial identification was made by using 16S rDNA sequencing. However, this technique may fail to differentiate genetically related species, as is the case for species belonging to the *C. novyi sensu lato* group [19]. This prompted us to perform NGS analysis, which confirmed that the isolate belonged to the species *C. haemolyticum*. It is noteworthy that identification of anaerobes has been significantly improved in clinical laboratories by the widespread use of the MALDI-TOF mass spectrometry. In our case, we used the two commercially available MALDI-TOF MS systems for identifying colonies obtained during the second bacteremic episode. Correct identification at the species level could be achieved using the Maldi Biotyper system, whereas the isolate remained unidentified when the Vitek MS system was used. This difference in identification performance is explained by the fact that, at the time when the case occurred, our laboratory was using the Vitek MS 2.0 database, which did not contain *C. haemolyticum* spectra. It should be noted that *C. haemolyticum* is now one of the species claimed in the new version of the database (v3.2) used by the Vitek MS.

In conclusion, this case illustrates the fact that, although rare in humans, infections caused by *C. haemolyticum*, are severe and should be considered as a potential factor in any febrile patient with hemolytic anemia. This case also highlights the importance of using molecular techniques for the identification of this fastidious anaerobic organism.

## Data Availability

The whole genome sequence of the isolate LBN 1021 (isolate 122-15, Institute Pasteur) is available at https://www.ncbi.nlm.nih.gov/assembly/GCA_910822285.1.
